# Smoking-attributable mortality in Morocco: results of a prevalence-based study in Casablanca

**DOI:** 10.1186/2049-3258-72-23

**Published:** 2014-07-01

**Authors:** Nabil Tachfouti, Chantal Raherison, Adil Najdi, Majdouline Obtel, Ahmed Rguig, Amina Idrissi Azami, Chakib Nejjari

**Affiliations:** 1 Laboratory of Epidemiology, Clinical Research and Community Health, Faculty of Medicine, Fez 30000, Morocco; 2 INSERM U897, ISPED, University Bordeaux Segalen, 33076 Bordeaux, France; 3 Department of Respiratory Diseases, CHU, Bordeaux 33604, France; 4 Department of Epidemiology and Disease Control, Ministry of Health, Avenue Ibn Sina, 10080 Rabat, Morocco; 5 Epidemiological Surveillance and Public Health Unit, Regional Direction of Health, Casablanca, Morocco

## Abstract

**Background:**

Tobacco control measurements’ had little impact on smoking prevalence in Morocco. The aim of this study is to provide first data on smoking attributable mortality in Morocco.

**Method:**

The Smoking-Attributable Mortality, Morbidity and Economic Costs (SAMMEC) software was used to estimate the smoking attributable mortality (SAM) in Casablanca region in 2012. Smoking prevalence and mortality data of people aged 35 years or older were obtained from the national survey on tobacco “Marta” and from Health Ministry Mortality System, respectively.

**Results:**

Of the 5261deaths of persons aged 35 years and older, 508 (9.7%) were attributable to cigarette smoking. This total represents 16.2% of all male deaths (n =448) and 2.0% (n =80) of all female deaths in this region. The leading four causes of smoking attributable deaths were lung cancer (177), chronic airways obstruction (76), ischemic heart disease (39), and cerebrovascular disease (31).

**Conclusion:**

Tobacco use caused one out of six deaths in Casablanca in 2012. Four leading causes (lung cancer, ischemic heart disease, cerebrovascular disease and chronic airways obstruction,) accounted for 51.6% of SAM. Effective and comprehensive actions must be taken in order to slow this epidemic in Morocco.

## Background

Scientific evidence of harm caused by smoking has been accumulating for over 200 years, at first in relation to cancers of the lip and mouth, and then in relation to vascular diseases and lung cancer [1]. Cigarette smoking has been identified as the second leading risk factor for death from any cause worldwide [2,3]. In 2000, an estimated 4.83 million deaths were attributed to cigarette smoking globally, with nearly half occurring in the developing world [4]. In people over age 30, smoking accounts for one in every five deaths among men and one in every 20 deaths among women globally [5]. The World Health Organization (WHO) has estimated that approximately 5.4 million people died worldwide from tobacco-related illnesses in 2006 and says that “unless urgent action is taken, tobacco’s annual death toll will rise to more than eight million” by the year 2030 [6]. Because many low- and middle-income countries are still in early stages of the tobacco epidemic, the number of smoking-related deaths in these nations will probably increase during the next decades. It is estimated that in the period 2002/2030, tobacco-attributable deaths will decrease by 9% in developed countries, but increase by 100% (to 6.8 million) in developing countries [7].

In contrast to deaths which are clearly attributable to a given factor, for example, accidental deaths, deaths due to smoking are harder to identify. The number of deaths caused by tobacco use in a population (the smoking-attributable mortality, SAM) can be estimated by different methodologies [8-10]. Peto’s ‘indirect’ method10 used lung cancer rates to retroactively estimate smoking prevalence [11]. Malarcher calculated attributable fractions adjusted for age and other potential confounders [12]. Thun used the Cox proportional hazard model, incorporating a wide array of potential confounders [13]. McNulty used smoking status reports from death certificates [14].

The population attributable risk (PAR) methodology is the most commonly used [8]. PAR incorporates the prevalence of smoking and the relative risk (RR) associated with various amounts of smoking [8-10]. Adult Smoking-Attributable Mortality, Morbidity and Economic Costs (SAMMEC), an online application developed by the Centers for Disease Control and Prevention (CDC), use attributable risk formulas to estimate the number of deaths from cancer, cardiovascular and respiratory diseases associated with cigarette smoking [15]. SAMMEC has been applied in United States and other countries such as Australia [16], Canada [17], Spain [18] Brazil [19], Israel [20], and Italy [21]. Counting and establishing the causes of deaths is a matter of concern for the public health community. Information on deaths is crucial to the planning, implementation and evaluation of public health programs at local, national and international levels. In developed countries, data on major health risk factors are regularly obtained from population surveys and morbidity specific registers such as those for cancer. Many developing countries have reasonably reliable data on mortality by cause, but lack population data on the prevalence of risk factors, such as smoking, which are essential to establish public health policy priorities. Attempts to produce indirect estimates are needed, because an important share of the global tobacco burden falls on developing countries, where 84% of the 1.3 billion current smokers reside [20].

The Kingdom of Morocco has a surface area of 710 850 km^2^ and is situated in the north west of Africa with a population of 29.8 million (Census 2004), with an average per capita monthly income of $1200. Tobacco control measurements’ and antismoking legislation had little impact on the prevalence of smoking. A study on cardiovascular risk factors conducted in 2000 found smoking rates of 17.2% (31.5% for men and 0.6% for women) [22]. In 2006, nationwide smoking-specific studies have been performed looking at prevalence and determinants. The overall prevalence of current smoking was 18.5% (31.5% for males and 3.1% for females) [23]. Among daily smokers, the proportion of household income spent on tobacco was around 30% [24]. Moreover, no data is available about mortality attributable to smoking.

In 2006, a large population-based survey was conducted in seven Moroccan administrative regions to assess prevalence, knowledge and attitude towards tobacco among Moroccan adult population [22-24]. The results of the survey allowed, along with other information, to estimate for the first time the number of smoking attributable deaths in Casablanca, the biggest city and economic capital of Morocco. Greater Casablanca is the largest economical region representing 12% of total population whose 91.6% are urban and whose population is homogenous. There are 1.7 million men and 63% of the population are less than 35 years old (mean age 25 years). Life Expectancy at birth is 67.1 years for men and 70.7 for women (Moroccan Health Ministry, 2003).

## Methods

### Modelization

After considering all the methods that could be used to estimate smoking attributable fraction (SAF) in Morocco, we decided to use the population attributable risk (PAR) method. Direct estimates of mortality cannot be made because there is a lack of longitudinal studies on the differential mortality of smokers, former smokers and non-smokers, necessary to provide RR estimates for smoking-related diseases and mortality. The smoking impact ratio (SIR) method proposed by Peto [11] requires lung cancer mortality rates in never smokers, which are not available in Morocco. SAMMEC method was used to calculate age-adjusted SAM rates for persons aged 35 years and older, using age, sex and cause specific mortality rates, current smoking prevalence by age group and sex (which is available for Morocco), and the American Cancer Society’s Cancer Prevention Study II (CPS-II) relative risks [25].

SAM is calculated for each cause of mortality using the following formula: SAM = OM × PAF; where OM is the observed (absolute) mortality, and PAF the population attributable fraction. The following equations were used to calculate the PAF:

$$\begin{array}{ll} \mathrm{PAF}= & \left(\left(p0+p1\times \mathrm{RR}1+p2\times \mathrm{RR}2\right)-1\right)/\\ & \left(p0+p1\times \mathrm{RR}1+p2\times \mathrm{RR}2\right); \end{array}$$

where p0, p1 and p2 represent the prevalence of non-smokers, smokers and ex-smokers, respectively. RR1 and RR2 refer to the risk of dying for smoking related pathologies of smokers and ex-smokers respectively compared to a baseline population of non-smokers.

### Data sources

#### Mortality data

The 2012 mortality data for 19 adult smoking-related diseases were drawn from the Mortality declaration registries in eight prefectures (administrative department) in Casablanca. Deaths were categorized by cause, sex and age group. Diseases were coded according to International Disease Classifications ICD 10 as shown in, Table 1[26]. Data on deaths from burns or second hand smoke were not included in the present study. Causes of death were distributed into three groups:

**Table 1:** **ICD 10 codes for smoking related diseases **[26]

**Disease category**	**ICD 10**
**Malignant cancers**	
Trachea, lungs, bronchi	C33–C34
Esophagus	C15
Stomach	C16
Pancreas	C25
Larynx	C32
Lips, oral cavity, pharynx	C00–C14
Neck of the uterus	C53
Kidney and renal pelvis	C64-C65
Urinary bladder	C67
Acute myeloid leukaemia	C92.0
**Cardiovascular diseases**	
Ischemic heart disease	I20-I25
Cerebrovascular disease < 35	I60–I69
Atherosclerosis	I 70
Aortic aneurysm	I71
Other arterial disease	I72-I78
Other cardiaq diseases	I25
**Respiratory diseases**	
Bronchitis, Emphysema	J40-J43
Chronic airway obstruction	J44–J46
Pneumonia, Influenza	J10-J18

•**Group I**: malignant tumors (lung-trachea-bronchus, lip-oral cavity-throat, esophagus, larynx, cervix, bladder and urinary tract, kidney and pancreas);

•**Group II**: cardiovascular diseases (ischemic heart disease and cerebrovascular disease in groups aged 35–64 years and >64 years);

•**Group III**: respiratory diseases (chronic bronchitis and emphysema).

### Smoking data

Smoking prevalence rates for adults aged 35 years or older were obtained from MARTA survey data [23,27-30]. It is a national cross-sectional study of a random sample of 9,195 individuals aged 15–90 years conducted in 2006. The sampling was performed with stratification by region, socioeconomic level, age and sex, taking into consideration the urban-to-rural ratios in each region. The country was divided into seven regions: central north region (Fez and surroundings), occidental region (Casablanca and surroundings), northwest region (Tangier and surroundings), eastern region (Oujda and surroundings). In each region, a prefecture (administrative division) was randomly chosen according to the size of the population. Smoking habit was defined according to the International Union Against Tuberculosis and Lung Diseases guide (Slama 1998). Respondents were classified as smokers if they had smoked at least 100 cigarettes until the date of the interview (daily smokers if they daily smoked and occasional smokers if they smoked on some days), ex smokers if they had smoked but had quit (for > 3 months), and nonsmokers if they had never smoked or had smoked fewer than 100 cigarettes until the date of the interview. We extracted smoking data in Casablanca region according to gender for adult population aged ≥35 years.

### Relative risk of mortality

SAMMEC application uses the American Cancer Society’s Cancer Prevention Study II (CPS-II) relative risks [24]. The CPS-II is an ongoing prospective study of 1,185,106 residents in United States, aged 30 years or over, for those who, in 1982, had never smoked regularly, and for those who were then current cigarette smokers [31]. US Center for Disease Control and Prevention (CDC) estimates for smokers and exsmokers are given in Table 2. It shows that despite smoking cessation leading to substantial reduce relative risk of mortality, ex smokers are still at a higher risk than never-smokers. The relative risk ratio of smokers versus ex-smokers ranges from one unit to 3.2 for the pathologies in question. Even if the relative risk is reduced with the passing of time since smoking cessation and even if it can match that of never-smokers, on average this population has a greater aggregated risk than that of never-smokers. Table 2 shows mortality relative risk for smokers and ex-smokers versus nonsmokers according to sex and diseases.

**Table 2:** Relative risk of death for smokers and ex-smokers comparing to nonsmokers

	**Males**		**Females**	
**Disease category**	**Current smoker**	**Former smoker**	**Current smoker**	**Former smoker**
**Malignant neoplasms**				
Lip, Oral cavity, Pharynx	10.89	3.40	5.08	2.29
Esophagus	6.76	4.46	7.75	2.79
Stomach	1.96	1.47	1.36	1.32
Pancreas	2.31	1.15	2.25	1.55
Larynx	14.60	6.34	13.02	5.16
Trachea, lung, bronchus	23.26	8.70	12.69	4.53
Cervix Uteri	0.00	0.00	1.59	1.14
Kidney and renal pelvis	2.72	1.73	1.29	1.05
Urinary bladder	3.27	2.09	2.22	1.89
Acute myeloid leukemia	1.86	1.33	1.13	1.38
**Cardiovascular diseases**				
Ischemic heart disease				
Persons aged 35–64	2.80	1.64	3.08	1.32
Persons aged 65+	1.51	1.21	1.60	1.20
Other heart disease	1.78	1.22	1.49	1.14
Cerebrovascular disease				
Persons aged 35–64	3.27	1.04	4.00	1.30
Persons aged 65+	1.63	1.04	1.49	1.03
Atherosclerosis	2.44	1.33	1.83	1.00
Aortic aneurysm	6.21	3.07	7.07	2.07
Other arterial disease	2.07	1.01	2.17	1.12
**Respiratory diseases**				
Pneumonia, influenza	1.75	1.36	2.17	1.10
Bronchitis, Emphysema	17.10	15.64	12.04	11.77
Chronic airway obstruction	10.58	6.80	13.08	6.78

Source: *Centers for Disease Control and Prevention,* Project “Smoking-Attributable Mortality, Morbidity, and Economic Costs (SAMMEC)”, https://apps.nccd.cdc.gov/sammec/show_risk_data.asp.

## Results

In 2013, a total of 5261 deaths of individuals aged 35 years and older (2767 males; 2494 females) were reported in Casablanca. From this total, missing information about cause of death accounted for 933 of death certificates, 1787 deaths were linked to smoking related diseases (993 men and 794 female) and were taking into account for the estimation of SAM. Cardiovascular disease caused 1178 deaths, cancer was responsible for 457 deaths, and respiratory disease for 152 deaths. Table 3 shows number of observed deaths according to mortality cause and sex. The prevalence of current and former smoking by sex and two age groups (35–64 years and ≥ 65 years) are shown in Table 4. Prevalence’s of smokers and ex-smokers are much higher among men, with the latter category being generally higher than the former one, especially among older adults. Moreover, there are no smokers among women aged 65 and older. Concerning amount of smoking, 15.6% of smokers do not smoke daily, and most of the remaining (75%) smoke more than ten cigarettes per day.

**Table 3:** Repartition of number of observed deaths due to smoking related disease according to sex in Casablanca (2012)

**Disease category**	**Observed mortality**		
	**Males**	**Females**	**Total**
Malignant cancers	309	148	457
Cardiovascular diseases	576	602	1178
Respiratory disease	108	44	152
Total	993	794	1787

**Table 4:** Proportion of current, former and nonsmokers according to gender and age groups in Casablanca (2006)

	**Males**			**Females**		
**Age category**	**Non smoker % (35% CI)**	**Current smoker % (35% CI)**	**Ex smokers % (35% CI)**	**Non smoker % (35% CI)**	**Current smoker % (35% CI)**	**Ex smokers % (35% CI)**
35 - 64	32.7 (28.8 – 37.6)	36.3 (31.6 – 41.2)	31.0 (26.5 – 35.8)	89.4 (85.4 – 92.6)	5.5 ( 3.3 – 8.8)	5.2 ( 3.1 – 8.4)
≥65 years	32.5 (18.6 – 49.1)	15.0 (5.7 – 29.8)	52.5 (36.1 – 68.5)	100.0 (100.0 – 100.0)	0.0 (0.0 – 13.2)	0.0 (0.0 – 13.2)
≥35 years	32.6 (28.4 – 37.4)	34.2 (29.9 – 39.0)	33.2 (28.6 – 37.6)	90.2 (86.6 – 93.1)	5.1 (3.1 – 8.1)	4. 7 (2.8 – 7.8)

Mortality attributable fraction (MAF) in men varied from 0.1 for cerebrovascular disease (person aged 65 years and older) to 0.91 for lung cancer and bronchitis and emphysema. In women, it was very lower especially for cardiovascular diseases; the highest SAF for women’s was around 0,44 fir lung and larynx cancers as shown in Table 5.

**Table 5:** Smoking attributable fraction according to sex and diseases

**Disease category**		**CID 10**	**SAF**	
**Malignant cancers**			**Male**	**Female**
Trachea, lungs, bronchi		C33–C34	0.91	0.43
Esophagus		C15	0.76	0.30
Stomach		C16	0.33	0.03
Pancreas		C25	0.33	0.08
Larynx		C32	0.87	0.44
Lips, Oral cavity, Pharynx		C00–C14	0.81	0.21
Neck of the uterus		C53	xxxx	0.05
Kidney, renal pelvis		C64-C65	0.46	0.02
Urinary bladder		C67	0.53	0.09
Acute myeloid leukaemia		C92.0	0.29	0.01
**Cardiovascular diseases**				
IschemCI heart disease	<35	I20-I25	0.46	0.12
	>35		0.16	0
Cerebrovascular disease	<35	I60–I69	0.46	0.15
	>35		0.10	0
Atherosclerosis		I 70	0.38	0.05
AortCI aneurysm		I71	0.71	0.27
Other arterial disease		I72-I78	0.27	0.07
Other cardiaq diseases		I25	0.26	0.04
**Respiratory diseases**				
Bronchitis. Emphysema		J40-J43	0.91	0.53
ChronCI airway obstruction		J44–J46	0.84	0.487
Pneumonia, Influenza		J10-J18	0.28	0.07

Of total 5261 deaths recorded in Casablanca in 2012 among person aged 35 years and older, 508 were attributed to smoking in the three groups of selected causes; 448 men’s and 60 women’s. Smoking accounted for 9.7% of all deaths; 16.2% of deaths in men, and 2.0% in women. Cancer was the most frequent cause, responsible for 247 of all smoking attributable deaths, followed by Cardiovascular diseases (160 deaths) and respiratory diseases (101 deaths).

The four leading specific causes of adult smoking attributable deaths were lung cancer (177 deaths: 159 men’s and 18 women’s), chronic airways obstruction (76 deaths; 62 men’s and 14 women’s), ischemic heart disease (39 deaths: 37 men’s and two women’s), cerebrovascular disease (31 deaths: 28 men’s and three women’s). Combined, these four conditions were responsible for 63.6% of all SAM (323/861); 64.0% among men and 61.6% among women’s. Table 6 presents the number of smoking-attributable deaths by sex grouped into three broad categories: cancer, cardiovascular and respiratory diseases.

**Table 6:** Observed mortality (OM) and smoking attributable mortality (SAM) according to sex and cause of death

**Disease category**			**Males**		**Females**		**Total**	
**Malignant cancers**		**CID 10**	**OM**	**SAM**	**OM**	**SAM**	**OM**	**SAM**
Trachea, lungs, bronchi		C33–C34	175	159	41	18	216	177
Esophagus		C15	5	4	2	1	7	5
Stamach		C16	25	8	25	1	50	9
Pancreas		C25	23	8	16	1	39	9
Larynx		C32	14	12	2	1	16	13
Lips, Oral cavity, Pharynx		C00–C14	11	9	2	1	13	10
Neck of the uterus		C53		====	38	1	38	1
Kidney, renal pelvis		C64-C65	4	2	1	0	5	2
Urinary bladder		C67	22	12	3	0	25	12
Acute myeloid leukaemia		C92.0	30	9	18	0	48	9
Subtotal			309	223	148	24	457	247
**Cardiovascular diseases**								
IschemCI heart disease	<35	I20-I25	30	14	16	2	46	16
	>35		146	23	165	0	311	23
Cerebrovascular disease	<35	I60–I69	36	17	21	3	57	20
	>35		110	11	116	0	226	11
Atherosclerosis		I 70	72	27	74	4	146	31
AortCI aneurysm		I71	4	3	3	0	7	3
Other arterial disease		I72-I78	33	9	36	3	69	12
Other cardiaq diseases		I25	145	38	171	6	316	44
Subtotal			576	142	602	18	1178	160
**Respiratory diseases**								
Bronchitis, Emphysema		J40-J43	18	16.	6	3	24	19
ChronCI airway obstruction		J44 – J46	74	62	30	14	104	76
Pneumonia. Influenza		J10-J18	16	5	8	1	24	6
Subtotal			108	83	44	18	152	101
Total			**993**	**448**	**794**	**60**	**1787**	**508**

Males and females differed slightly in the ranking of the four leading causes of smoking attributable deaths. Among males they were: lung cancer (159 deaths), chronic airways obstruction (62 deaths), ischemic heart disease (IHD) (37 deaths), and cerebrovascular disease (31 deaths). Among females they were lung cancer (18 death), chronic airways obstruction (14 deaths), atherosclerosis (38 deaths), cerebrovascular disease (three deaths). Table 6 shows Observed mortality (OM) and smoking attributable mortality (SAM) according to sex and related smoking cause of death.

## Discussion

To our knowledge, this is the first study to estimate SAM in Morocco; cigarette smoking was responsible for 9.7% of all adult deaths (16.2% in men’s and 2.0% in women’s) and 28.4% of smoking related disease deaths (45.1% among men’s and 7.6% among women’s) in the studied population. The current SAM reported in this paper shows clearly how hazardous and costly in lives smoking are to a society. Combining the four leading causes of smoking attributable deaths in Casablanca cities in 2012; lung cancer, ischemic heart disease, cerebrovascular disease and chronic airways obstruction; account for 63.6% of the SAM. These diseases are among the most important causes of death in the country. In 2010, according to Health Ministry statistics, cardiovascular diseases, cancer and respiratory diseases together were responsible for 45.8% of all adult deaths in Morocco. Concerning cancer deaths, our results are in concordance with Casablanca cancer registry data (2005–2007) [32]. Incidence data show that among men lung localization represents 22.7% of all cancer localization, neck of uterus represents 13.3% of total female cancer localization. Thus, these results suggest that a large proportion of these deaths would be prevented by further reductions in smoking prevalence.

Smoking attributable fraction in men is 6 times higher comparing to women’s, because the female prevalence of smoking is 1/7 that of males and RR are similar for several disease. Tobacco was responsible for 9.7% of total deaths among person aged 35 years and older with an equal male-to-female SAM ratio. It is lower than proportion reported in Argentina (16%) [33], Portugal (11.7%) [34], Italy (12.5%) [21] and Brazil (13.6%) [19]. Moreover, in these countries, the most frequents cause of SAM are Lung cancer, Ischemic heart diseases and chronic obstructive airways, representing around more than 50% of total SAM. In South Africa, also a developing country, smoking accounted for 8.0 to 9.0% of deaths, with three times as many deaths occurring in males compared with females [35]. Our finding is lower than rate reported in Taiwan where 1 out of 4 deaths (27%) in middle aged men (35–69 years old) were attributable to smoking, smoking rate for adult males in 2001 was 47.3% [36]. Differences in SAM largely reflect the stage of the smoking epidemic in each country [37], but the above data show that the number of cigarettes smoked per day also plays a role. The SAM in Mexico in 2004 is much lower than the one observed in this work (5.2% of total deaths; 6.0% in men and 4.3% in women) [38]. Because prevalence of smoking in Mexico is higher than in Morocco, such difference in SAM is likely to reflect, at least in part, to the lesser amount of cigarettes smoked daily in Mexico [23].

However, some limitations of the survey need to be discussed. To estimate smoking attributable mortality, we used the SAMMEC software. This method uses present smoking exposure without considering the changing trend of smoking and latency of mortality causes. In fact, for most tobacco related diseases, smoking attributable deaths reflect smoking exposure in previous decades, since latency of lung cancer, other cancers and non-neoplasic respiratory diseases is of several decades [39]. Thus, the smoking attributable mortality estimates do not represent the past or cumulative smoking of the population of interest, but only reflect the current smoking profile [40]. A major British study [41] that showed the full effects of tobacco on national mortality rates can take more than 50 years to mature. Another limitation of the SAMMEC methodology is that it assumes RR estimates from CPS II. Although this represents one of the largest and best conducted studies to provide RRs of mortality according to smoking status, the validity of applying the RRs of a US population to the Moroccan one is open to discussion. Smoking histories, including in particular intensity and duration, and tobacco product usage of the CPS II participants might in fact differ from the Moroccan one, thus influencing the RRs of various tobacco-related diseases [42]. Environmental factors interacting with smoking are different in Morocco, compared to the U.S such as levels of outdoor pollution and indoor air pollution from cooking could modify the effects of smoking in Moroccan population in a non-multiplicative way [11]. Additionally, effect of potential confounders such as alcohol (in relation to some cancers) was not taking into consideration to estimate SAM.

The list of smoking-attributable diseases in SAMMEC does not include colorectal cancer among the malignancies. Studies in various populations show an association between tobacco use and colorectal cancer [43,44]. Recent Nurse’s Health Study evidence found that current smoking was associated with an increased risk of colorectal cancer mortality [45]. The addition of colorectal cancer to the list of tobacco-associated malignancies would further increase the SAM estimates presented in our work. In addition, two recent meta-analyses discuss the potential association between smoking and mortality from tuberculosis (TB) [45,46], but this contribution is still controversial, so TB has not been included in the present study. The potential limitations notwithstanding, the SAMMEC method had the advantage of allowing relatively fast computation, and enabling comparisons of our estimates with similarly produced estimates in other countries.

Information about mortality cause was not available from 17.7% of certificate of deaths which were not taking into account for total estimation suggesting that our finding underestimates the SAM. Mathers *et al.* analyzed the death registration system of 115 countries to determine the percentage of causes of deaths coded as unknown and ill-defined [47]. Based on these results, data quality for Morocco was categorized as low. However, the Moroccan Mortality System is far better in Casablanca and has improved considerably in recent years; we showed that the completeness of data is around 80%. Thus, the estimates of SAM in the city are likely to express the actual number of smoking attributable deaths.

Despite these limitations, our results show the importance and the priority to be accorded to measures to reduce the rate of smoking in the Moroccan population. Reducing smoking prevalence would have an important impact on SAM. A study in Taiwan has demonstrated that if the annual smoking rate were to be reduced by 10% between 2001 and 2020, the corresponding projected SAM would decrease by 30%.

A tobacco control law (law 15–91) was enacted in Morocco in 1996 [48]; it included a ban on advertising, a ban on sponsorship, and a ban on smoking in public transport and in educational and healthcare facilities. Officially launched on March 2010, the National Cancer Prevention and Control Program listed up 6 operational measures on tobacco control.

## Conclusion

With Morocco now moving towards ratifying the WHO Framework Convention on Tobacco Control (FCTC) which contains comprehensive anti-smoking policies [49], the need to adapt and enforce effective measure becomes crucial; especially in the following ways:

•More widespread enforcement of current legislation;

•A more complete tobacco control programme in light of current prevalence and upward trends in smoking;

•Reinforcing School-based tobacco use prevention interventions which are effective in reducing smoking prevalence, reducing smoking initiation and intended smoking intentions in the short term [50];

•High priority on ensuring that the tobacco industry does not undermine current and future regulations, and in future, implementation of FCTC obligations.

## Competing interests

The authors declare that they have no competing interests.

## Authors’ contributions

NT performed the statistical analysis and wrote the manuscript. CR corrected the manuscript. AN and MA participated in statistical analysis. AR and AIA coordinated data collection. CN performed protocol and coordinated study. All authors read and approved the final manuscript.
